# Thyroid Receptor Antibody and the Development of Graves’ Orbitopathy: Clinical Experience of using Radioiodine Ablation in the Management of Graves’ Orbitopathy in post-iodine ablation hypothyroid patient

**DOI:** 10.22038/AOJNMB.2023.68546.1478

**Published:** 2023

**Authors:** Edelyn Christina, Hendra Budiawan, Hapsari Indrawati, Erwin Affandi Soeriadi, Trias Nugrahadi, A Hussein Kartamihardja

**Affiliations:** 1 Department of Nuclear Medicine, Padjadjaran University, Bandung, Indonesia; 2 Department of Nuclear Medicine, Mochtar Riady Comprehensive Cancer Center Siloam Hospital, Jakarta, Indonesia; 3 Department of Nuclear Medicine, Hasan Sadikin Central Hospital, Bandung, Indonesia

**Keywords:** Graves’ orbitopathy, Graves’ ophthalmopathy, Radioactive iodine, Thyroid receptor antibody

## Abstract

Graves’ disease (GD) is the commonest cause of hyperthyroidism, accounted for 70-80% in iodine sufficient countries and up to 50% in iodine deficient countries. Combination of genetic predisposition and environmental factors influences the development of GD. Graves’ orbitopathy (GO) represents the most common extra-thyroidal manifestation of GD with substantial impact on morbidity and quality of life. Expression of thyroid stimulating hormone receptor (TSHR) mRNA and protein in orbital tissues infiltrated by the activated lymphocytes produced by thyroid cells (Thyroid Receptor Antibody) results in the secretion of inflammatory cytokines that leads to the development of histological and clinical characteristics of GO. A subdivision of TRAb, thyroid stimulating antibody (TSAb), was found to have a close relationship with the activity and severity of GO, and suggested to be considered as a direct parameter of GO. Here, we present a 75-year-old female with a history of GD that has successfully been treated with radioiodine treatment, who developed GO 13 months after therapy while being hypothyroid with high TRAb level. The patient was given a second dose of radioiodine ablation to maintain GO with successful result.

## Introduction

 Graves’ disease (GD) is the commonest cause of hyperthyroidism, accounted for 70-80% in iodine sufficient countries and up to 50% in iodine deficient countries. It is typically presented in patients between 40-60 years old, and predominantly affects women, with female to male ratio was estimated around 8:1(1). The combination of genetic predisposition and environmental factors (2, 3) influences the development of GD. Using structural equation model to test the effect of genetic and environmental components in the development of GD amongst twin subjects, Brix et al. were able to estimate the 80% attributability of genetic factors and 20% of individual-specific environmental factors (3). Environmental factors that are postulated to affect the development of GD are iodine, medications, infections, smoking, as well as stress (2).

 The immunopathogenesis of GD is established. Thyrotropin receptor antibodies (TRAb) produced by B-cell clones, stimulates the thyroid stimulating hormone receptor (TSHR) in a manner that resembles TSH and further induce thyrocytes proliferation, thyroid growth, and secretion of thyroid hormones (4,5). GD is characterized by hyperthyroidism, diffuse hyperfunctioning goiter and may be accompanied by extra-thyroid manifestation such as orbito-pathy, myxedema and acropachy. 

 Graves’ orbitopathy (GO) represents the most common extra-thyroidal manifestation of GD with substantial impact on morbidity and quality of life (6,7). A study in Minnesota previously estimated GO to contribute to 16/100,000 per year for women and 2.9/100,000 per year for men (8). Several reports have been published to confirm the expression of TSHR mRNA and protein in orbital tissues in healthy individuals and those with GO (7, 9). Infiltration of the orbital tissue by activated lymphocytes results in the secretion of inflammatory cytokines that leads to the development of histological and clinical characteristics of GO. GO may develop prior or after the onset of hyperthyroidism, with only 20-30 % GO patients require aggressive treatments (7).

***Case Report***

 A 75-year-old female presented with swollen and teary eyes, headache, and intermittent discomfort. She was diagnosed with graves’disease three years earlier and was given methimazole with no improvement. She had undergone radioactive iodine ablation with 555 MBq (15 mCi) of I-131 6 months after she was given methimazole, and achieved hypothyroid state 4 months following radioactive iodine therapy. Orbital symptoms reappeared 13 months following radioactive iodine ablation. Blood pressure was 120/80 mmHg, and pulse was regular, full volume, with 70 beats per minute. Thyroid gland was not palpable on physical examination. 

 Free thyroxine level and TSHs level under the influence of levothyroxine 16 months following the first radioactive iodine ablation were 1.39 ng/dL (normal value: 0.93–1.71 ng/dL) and 10.1µIU/mL (normal value: 0.27–4.2 µIU/mL), respectively. TSH receptor antibody (TRAb) was 7.07 IU/L (normal value <1.75 IU/L). She took levothyroxine 100 µcg once a day, selenium 100mg two times a day, and three weeks of methyl prednisolone tapered off from 3×16mg, to 2×16mg in the second week, and 1×16mg in the third week. Orbitopathy symptoms subsided and remained stable for 7 months after steroid therapy, until swollen eyes reappeared, accompanied with teary eyes, headache, double vision, and eyes discomfort. Clinical activity score was 3, each accounted for painful feeling behind the eyes, pain upon gaze, and eyelid swelling. Other than that, patient also complained bulging of both eyes. Hertel’s measurement was not performed to assess the proptosis.

 On follow up, free thyroxine level and TSHs level under the influence of levothyroxine were 1.71 ng/dL (normal value: 0.93–1.71 ng/dL) and 1.88 µIU/mL (normal value: 0.27–4.2 µIU/mL), respectively. TRAb was 7.35 IU/L (normal value <1.75 IU/L). Head magnetic resonance imaging (MRI) shows relatively symmetrical hypertrophy of bilateral extra ocular muscles, and mild hypertrophy of bilateral lacrimal glands. She took levothyroxine 50 µcg once a day, selenium 100mg two times a day, vitamin D 1×5000 IU, and another course of three weeks of methylprednisolone. She was asked to stop levothyroxine and undergo thyroid scintigraphy with technetium-^99^m pertechnetate one month after she stopped taking levothyroxine. Thyroid scintigraphy showed residual thyroid uptake on both glands (Figure 1, Figure 2).

**Figure:**
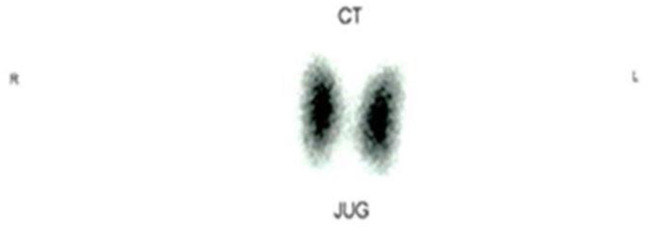


**Figure:**
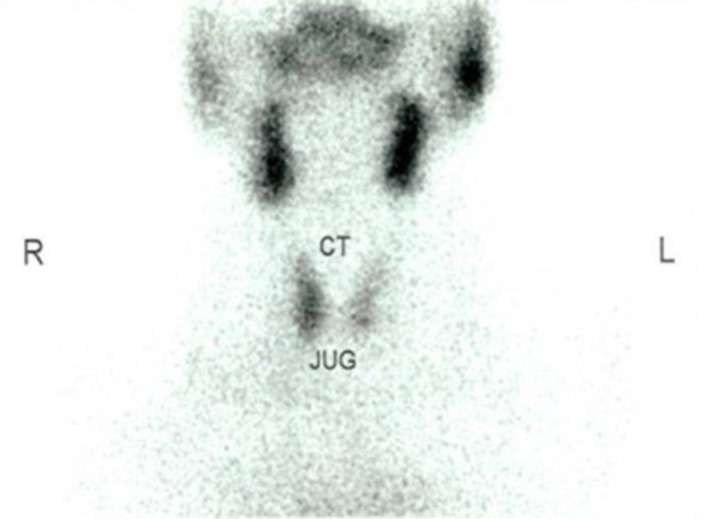


 Radioactive iodine as much as 185 MBq (5 mCi) was given for remnant thyroid ablation whilst being hypothyroid and no orbitopathy symptoms. Free thyroxine level and TSHs level were 1.45ng/dL (normal value: 0.93–1.71 ng/dL) and 14.6 µIU/mL (normal value: 0.27–4.2 µIU/mL), respectively three months following the second radioactive thyroid ablation. TRAb was <0.9 IU/L (normal value <1.75 IU/L). Orbitopathy symptom improved significantly and remained stable until almost one year after the second administration of radioactive iodine therapy. 

## Discussion

 The involvement of TSHR as the target antigen of autoimmune response in graves hyperthyroidism is established. Interestingly, the presence of TSHR on orbital fibroblasts have been found to be significantly higher in patients with GO compared to healthy individuals (10). TSHR antibody titer was also found to be correlated with the clinical activity of GO (11). These findings support the hypothesis of TSHR as the target antigen of GO. Orbital inflammation in GO may be initiated by infiltrating T-helper cells in the periorbital area, leading to production of cytokines, free oxygen radicals, and TRAb, and further induce fibroblast proliferation and glycosaminoglycan production (10). Alternatively, cross reactivity involving recognition of antigenic TSHR peptide within the thyroid by lymphocytes stimulate the production of TRAb that binds to the TSHR on orbital fibroblasts which later leads to the development of GO (12)(Figure 3).

**Figure:**
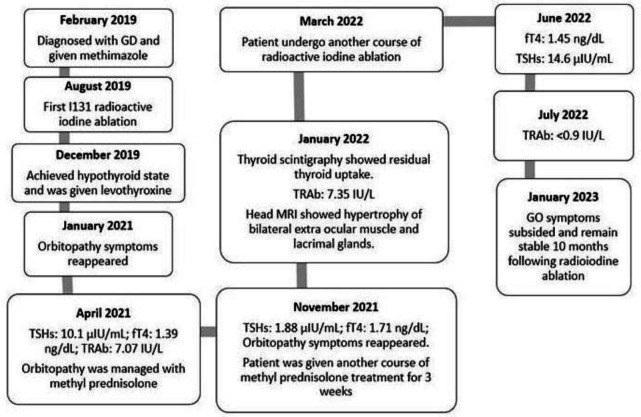


 According to the functional effect, TRAb are subdivided into thyroid stimulating antibody (TSAb), thyroid blocking antibody (TBAb), and neutral antibody. Amongst the three antibodies, TSAb and TBAb have been found to be associated, with the development of GO (11). A study evaluating the relationship between TSAb or TBAb and the clinical changes of GO in 155 untreated patients found a significant relationship between TSAb and clinical characteristics of GO, such as extra ocular muscle enlargement and increment of orbital fat (13). Systematic review of 26 studies also found that TSAb is positively correlated with GO, and a better indicator of GO in post-treated GD patients compared to TBAb (14).

 Therapeutic ablation using radioactive iodine (RAI) to eliminate thyroid antigen have been used to treat GD for many years. Treatment of GD using RAI is followed with changes in thyroid autoimmunity that results in transient increase of TRAb level. Radiation-induced thyroid damage, leading to the release of thyroid antigens that further increase the production of thyroid autoantibodies has been proposed to cause the change of TRAb level (15). This change in TRAb has been observed to start immediately after RAI therapy and achieved maximal value at 3 months after therapy.

 The surge of TRAb after RAI was blamed to be the reason of GO exacerbation. In line with this hypothesis, a recent randomized clinical trial assessed the effect of different doses (fixed low dose, fixed high dose, and calculated dose) of RAI in the development of GO after therapy showed that patients who received calculated dose of I-131as much as 5.55MBq (150 µCi)/g of thyroid weight had significantly lower amount of CAS difference before and after therapy compared to patients who received fixed high dose (Mean±SD were -0.03±0.4 vs 0.34±0.6, respectively with p value: 0.006), with comparable therapeutic response 12 months following therapy. This leads to interesting hypothesis that well-controlled thyroid ablation achieved through dose calculation method may prevent uncontrolled release of antigens with limited immune reaction (16). In this patient however, we did not perform thyroid weight calculation due to limited resources in our department. Our patient received 555MBq (15mCi) of RAI, which was slightly lower than the highest amount given to the patient in calculated dose group in the study (580.9 MBq). Despite that, thyroid scintigraphy performed more than two years following RAI ablation still showed functional thyroid glands with high TRAb level, even while patient taking thyroid hormone to maintain her hypothyroid state.

 Early thyroxine administration after RAI ablation was previously described to reduce the development of GO in a retrospective study, perhaps through its direct action on B lymphocyte or inhibition of antigenic substances production by the thyroid. In the study, the authors gave thyroxine 2 weeks following RAI therapy, while patients were still hyperthyroid. Interestingly, when compared to patients who were given thyroxine after serum concentration of TSH and/or T4 indicated hypothyroidism, the number of patients whose ophthalmopathy symptoms developed or worsened were higher in this group (27 patients (11%) vs. 45 patients (18%) in patients with and without early thyroxine administration, respectively; p value: 0.04; RR: 1.64 (95%CI=1.05-2.55))(17). Our patient was given levothyroxine after hypothyroidism occurs, as recommended by American Thyroid Association (2016) (18). Although this finding is interesting, to our knowledge, there has not been any carefully designed prospective clinical trial to prove this hypothesis.

 Recent retrospective study aimed to determine the incidence of GO in 76 patients following RAI treatment found a slight insignificant increase of mean CAS score in 2 months compared to 1 year after RAI treatment (Mean[SD]: 0.63[1.02] and 0.83[1.01]; p value: 0.174, respectively) (19). Nevertheless, progression of GO can also be observed after near total thyroidectomy (20, 21). This observation suggests that the persistence of thyroid tissues, even for minimal amount, could induce the activation of antigen-autoimmune cross-reactivity of thyroid and orbital tissues. Previous study using RAI modality for the treatment of GO in patients with hyper-,eu-, and hypo-thyroid, with nine of whom were hypothyroid, described successful outcome in the majority of patients (11 out of 15) (22). 

 Another study using RAI treatment for the ablation of total thyroid remnant after near total thyroidectomy also described significant increase in the number of patients with inactive GO after RAI treatment. The prevalence of inactive eye disease was significantly higher amongst patients received RAI after near total thyroidectomy compared to patients received near total thyroidectomy alone (87.5% and 71.8%; p value<0.0005, respectively) (23). 

 Thus, residual thyroid tissues in post-RAI hypothyroid patients may also contributes to antigen-antibody cross reactivity between thyroid and orbital tissues.

 Our patient had her eyes symptoms worsened long after being hypothyroid. Attempt to relieve symptoms using oral steroid medication only alleviate symptoms for seven months until reappeared. According to symptoms, the presence of proptosis, as well as CAS value, the patient was at least classified as mild-to-moderate GO according to European Group on Graves’ Orbitopathy (EUGOGO) classification (24). These apparent signs and symptoms were subsided completely following the second course of steroid medication, which followed by second RAI ablation. Although the patient did not complain about any specific sign or symptom following the second steroid medication, head MRI reveal hypertrophy of bilateral extraocular muscles and lacrimal glands. She was offered another course of steroid medication to pacify this finding, but declined due to concern of side effects. The decision for second RAI ablation was considered because TRAb level was high and showed increasing trend albeit not significant, accompanied by apparent uptake of technetium-^99^m pertechnetate on thyroid scintigraphy. TRAb level was significantly reduced within four months following the second RAI ablation without any symptoms.

 Remnant thyroid tissues ablation using RAI may not be the ideal approach, especially considering the patient needs to stop thyroxine hormone, which render hypothyroidism. Radioiodine therapy has been reported to cause the aggravation of GO, given the surge of TRAb level following its administration (15). Careful calculation of RAI dose based on thyroid weight of each patient during the first RAI ablation might be a better alternative to limit the incidence of GO after RAI ablation (16). 

 Nevertheless, the use of RAI treatment in the management of GO might be useful in patients with apparent functional thyroid remnant that induce antibody production against TSHR. We speculate that RAI treatment works by depriving the antigen. It should be noted however; that the use of RAI treatment in the management of graves’ orbitopathy, especially with hypothyroidism, have not been studied in a prospective controlled study.
